# Nanoimprint Lithography for Next-Generation Carbon Nanotube-Based Devices

**DOI:** 10.3390/nano14121011

**Published:** 2024-06-11

**Authors:** Svitlana Fialkova, Sergey Yarmolenko, Arvind Krishnaswamy, Jagannathan Sankar, Vesselin Shanov, Mark J. Schulz, Salil Desai

**Affiliations:** 1 NSF Engineering Research Center for Revolutionizing Metallic Biomaterials, North Carolina A&T State University, Greensboro, NC 27411, USA; sfialkov@ncat.edu (S.F.); sergey.yarmolenko@gmail.com (S.Y.); sankar@ncat.edu (J.S.); 2 College of Engineering and Applied Sciences, University of Cincinnati, Cincinnati, OH 45221, USA; krishnaswamyarvind1@gmail.com (A.K.); shanovvn@ucmail.uc.edu (V.S.); schulzmk@ucmail.uc.edu (M.J.S.); 3 Center for Excellence in Product Design and Advanced Manufacturing, North Carolina A & T State University, Greensboro, NC 27411, USA

**Keywords:** carbon nanotubes, chemical vapor deposition, nanoimprint lithography, magnetron sputtering, reactive ion etching

## Abstract

This research reports the development of 3D carbon nanostructures that can provide unique capabilities for manufacturing carbon nanotube (CNT) electronic components, electrochemical probes, biosensors, and tissue scaffolds. The shaped CNT arrays were grown on patterned catalytic substrate by chemical vapor deposition (CVD) method. The new fabrication process for catalyst patterning based on combination of nanoimprint lithography (NIL), magnetron sputtering, and reactive etching techniques was studied. The optimal process parameters for each technique were evaluated. The catalyst was made by deposition of Fe and Co nanoparticles over an alumina support layer on a Si/SiO_2_ substrate. The metal particles were deposited using direct current (DC) magnetron sputtering technique, with a particle ranging from 6 nm to 12 nm and density from 70 to 1000 particles/micron. The Alumina layer was deposited by radio frequency (RF) and reactive pulsed DC sputtering, and the effect of sputtering parameters on surface roughness was studied. The pattern was developed by thermal NIL using Si master-molds with PMMA and NRX1025 polymers as thermal resists. Catalyst patterns of lines, dots, and holes ranging from 70 nm to 500 nm were produced and characterized by scanning electron microscopy (SEM) and atomic force microscopy (AFM). Vertically aligned CNTs were successfully grown on patterned catalyst and their quality was evaluated by SEM and micro-Raman. The results confirm that the new fabrication process has the ability to control the size and shape of CNT arrays with superior quality.

## 1. Introduction

Carbon nanotubes (CNTs) in their varied forms have had a profound impact on the fabrication of next-generation devices [1,2,3,4]. These include single walled and multiwalled carbon nanotubes. Further, single walled carbon nanotubes can exist in armchair, zigzag, and chiral configurations [5,6]. Several methods have been devised for the synthesis and fabrication of CNT-based devices [7,8,9]. Many products using CNTs today incorporate CNT powders dispersed in polymer matrices or deposited as thin films in order to commercialize these products [10,11,12,13,14]. Organized CNT materials such as forests and yarns are beginning to bridge the gap between the nano-scale properties of CNTs and the length scales of bulk engineering materials [15,16,17,18]. However, the properties of CNT yarns and sheets, such as thermal conductivity and mechanical strength, remain far lower than the properties of individual CNTs [19,20,21,22]. Thus, the placement of individual CNTs having the desired structure with lithographic precision over large substrates would be a breakthrough for electronic devices [23,24,25,26,27]. So far, several successful attempts have been achieved in controlling the size and location of CNT arrays at the micro-level: Y. H. Yun et al. [10] reported on a multiwalled carbon nanotube (MWCNT) tower electrochemical actuator. Using the optical lithography method, his group developed a high-sensitivity electrode with a size of 100 microns and used it for cancer cell detection [10,15,19,28]. The approach of patterning the catalyst on a substrate and then synthesizing CNT was attempted earlier, wherein the CNT arrays were prepared by the pyrolysis of iron phthalocyanine that were patterned by UV NIL substrates. The lateral dimension of arrays was 20 microns [29]. Similar results were obtained by A.J. Hart, where the aligned CNT arrays were synthesized on patterned substrate and post-processed with organic solvent to shrink and improve alignment within individual bundles of CNTs [30,31]. Kim et al. fabricated 3D carbon nanostructures with sizes below 1 micron [18,22]. A vertically formed and hexagonally aligned nano-scale tubular carbon array was fabricated through carbon deposition inside an anodic aluminum oxide nano-template, followed by controlled chemical etching of the alumina layer. Nano-scale carbon pillars with controlled diameters, down to 25 nm, and protruded lengths of 100 nm were successfully used as a master-mold for UV-NIL [32]. Nanoimprint lithography (NIL) [33,34,35,36,37] is a versatile nanofabrication technique that can pattern nanoscale features with high fidelity resolution on a variety of substrates in combination with thin-film deposition processes [38,39,40,41,42]. Thus, it is a viable processing method to incorporate catalyst for the growth of CNTs with a high aspect ratio. Depending on the type of resist being employed, NIL can build features down to 20 nm with 3-dimensional topographies. 

Ongoing interest in CNTs as components of biosensors [43,44,45] and medical devices [46,47,48] is motivated by the dimensional and chemical compatibility of CNTs with biomolecules, such as DNA and proteins [29,49,50,51,52,53]. In addition, the unique mechanical, electrical, thermal and optical properties of CNTs enable fluorescent and photo-acoustic imaging, as well as localized heating using near-infrared radiation [54,55,56,57]. Singe walled carbon nanotubes (SWCNT) biosensors can exhibit large changes in electrical impedance and optical properties in response to the surrounding environment [58,59,60]. Low detection limits and high selectivity require engineering the CNT surface modification by functional groups and coatings and appropriate sensor design. Similar CNT sensors have been used for gas and toxin detection in the food industry, military, and environmental applications [13,61,62,63,64,65,66,67].

Nanoimprint Lithography (NIL) has been used by several researchers to pattern pre-synthesized CNTs onto substrates [24,50,51]. However, in our research, we focus on the growth of CNTs using NIL based on two different growth routes. The Process A (negative patterning) route focuses on using NIL to pattern resist followed by etching to create channels that are deposited with Fe catalyst by magnetron sputtering. However, Process B (positive patterning) aims to pattern a uniformly deposited Fe catalyst layer using an NIL resist pattern. We investigate both processing conditions and subsequent growth mechanisms in CNT growth. Thus, though NIL has been used in the past to pattern different nanomaterials [68,69] such as CNTs, quantum dots, and amorphous materials, our research explores the use of NIL for growth of CNT islands. 

The CNTs grown by NIL patterning in this research can be used in different formats including CNT tufts, CNT spun into threads, and CNT dispersed in colloidal media, as well as layer-by-layer self-assembly in a thiol-based solution [70,71]. The aim is to create high aspect ratio CNTs without significant entanglements among themselves to aid dispersion in media for energy storage [72], thermal barrier materials [73], molecular electronics [74], structural composites [75], biomedical sensors [76] and catalyst supports [77].

This research aims to create novel 3D carbon nanotube materials/devices by modifying the catalyst surface at the nano-level using a combination of technologies: Magnetron Sputtering Deposition and Nanoimprint Lithography. The ability to control the shape and size of catalyst gives the ability to manipulate the shape of CNTs arrays, sizing them to the required dimensions, and placing them in a specified location. Thus, large-scale arrays of CNTs can be reliably grown without entanglement during their processing. 

## 2. Materials and Methods

### 2.1. Patterning Catalyst System

**Figure 1:**
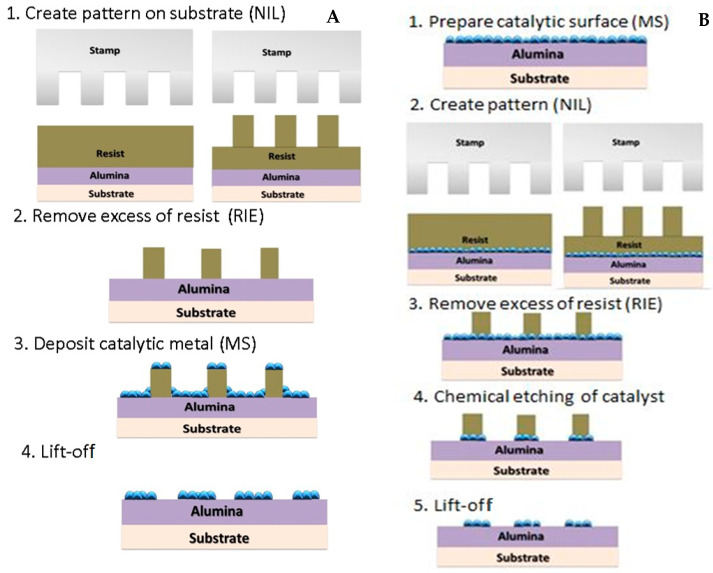
Catalyst patterning processes: (**A**) “negative” and (**B**) “positive” pattern replica/transfer.

The basic scheme of steps required to prepare the patterned catalyst is presented in Figure 1. Silicon (Si) substrate was used to deposit alumina coating as the base layer on which the resist materials were coated. The procedure includes processes of: resist spin-coating and imprint; plasma etching to remove the residual layer of resist and open substrate surface; deposition of alumina and catalytic metal; chemical etching of catalyst metal; and the final step of lift-off of thermal resist residue. Depending on the sequence of steps, the positive or negative replica of the master–stamp (mold) pattern can be replicated. To achieve repeatable results, each process step was optimized [78].

In Process A (Figure 1A), the photopolymer resist was deposited in stage 1, followed by nanoimprinting of patterns. Reactive ion etching was performed to etch away the resist material through the Si substrate. Further, magnetron sputtering was used to deposit the alumina and catalytic iron material, which was followed by the removal of the residual resist material using lift-off. In this paper, Process A is defined as “Negative” as only the areas not covered by the nanoimprinted photoresist are deposited by the alumina and catalytic iron material.

In Process B (Figure 1B), magnetron sputtering was used to deposit the alumina and catalytic iron material on the entire Si substrate in stage 1. This was followed by deposition of the photopolymer resist and subsequent nanoimprinting of patterns. Reactive ion etching was performed to etch away both the (alumina + catalytic iron material) and patterned resist material. Further, the residual resist material on top of the catalytic iron metal was removed using lift-off. In this paper, Process B is defined as “Positive” as the entire substrate was deposited with alumina and catalytic iron material.

Both approaches described above were implemented to evaluate differences in patterning transfer resolution, catalytic material deposition, and growth of CNTs. The line widths of the deposited catalyst depend on the etching profiles of the nanoimprinted features. REI may result in undercuts or orthogonal etching profiles of the resist materials resulting in lower or less accurate line width of features, respectively. In the case of undercut etching profiles for Process A, the subsequent deposition of alumina and catalyst material can result in slightly oversized line widths. Contrastingly, undercut etching profiles for Process B can result in lower line widths. These outcomes are explained in the Results Section.

### 2.2. Thermal Resists

Thermal resists supplied by Nanonex—NRX1025 2.5% and 7% and polymethyl methacrylate (PMMA) mr-35k from Sigma Aldrich (St. Louis, MO, USA) were used for this research. The resists NRX1025 2.5% and 7% and PMMA mr-I 35k were spin-coated on a Si substrate and on 10 nm alumina layer deposited by RF sputtering with a roughness of Ra = 0.2 nm. The resists were spin-coated at various speeds from 2000 rpm to 6000 rpm, and the substrates were baked on a hot plate at 150 °C for 5 min to remove the residue of solvent. The lift-off of the resist was conducted in an ammonia-hydrogen peroxide solution and rinsed with acetone.

### 2.3. Nanoimprint Lithography (NIL)

The nanoimprint lithography tool Nanonex 2000 (Nanonex, Monmouth Junction, NJ, USA) was used in the thermal mode to create the patterns on the substrate. The system is designed to provide consistent and uniform pressure across the surface of a sample (up to a 4″ wafer). Nanonex 2000 is specially designed with an air-cushion press which levitates the substrate and mold assembly providing a self-aligning feature, thereby preventing localized high stress regions and breakage of the nanoimprint assembly components.

### 2.4. Magnetron Sputtering

An AJA International Model ATC 1800F (AJA International, Hingham, MA, USA) magnetron sputtering system was used for the deposition of an alumina support layer and an iron catalyst on Si/SiO_2_ substrates (Silicon Quest, San Jose, CA, USA). The system has a deposition chamber with a base pressure of under 10^−7^ mTorr, a load-lock chamber and programmable gas flow controllers. The AJA International magnetron sputtering system is composed of three targets and can be used to perform radio frequency (RF) sputtering, direct current (DC) sputtering or pulsed-direct current (PDC) sputtering.

### 2.5. Reactive Plasma Etching

Reactive plasma etching (PE-100, PlasmaEtch, Carson City, NV, USA) was used to reduce the thickness of the spin-coated films for catalyst deposition. The effects of gas pressure, applied power, and oxygen content in the reactive gas mixture were evaluated.

### 2.6. Characterization Tools

Atomic force microscopy (MFP-3D Origin, Asylum Research, Goleta, CA, USA) and scanning electron microscopy (Hitachi S-4800, Hitachi, Tokyo, Japan) were used to measure the three-dimensional features for characterization. The thickness of the nanoimprinted resist was measured by the XRD technique and a profilometer. For the XRD thickness measurements, X-ray beams were diffracted at different angles θ of the deposited thin-film sample by a highly collimated X-ray beam [79,80]. The average film thickness was calculated from the constructive interference of the diffracted beams of the interplanar spacing of the crystalline material based on the Bragg law. Raman analysis and EDS spectra were used to characterize the CNT formations including their multiwall structures. Scanning electron microscopy was used to characterize specific deposited patterns of both the catalyst and the final CNTs.

### 2.7. Chemical Vapor Deposition (CVD)

The EasyTube^TM^ 3000EXT system (FirstNano, Islip, NY, USA) was used for chemical vapor deposition (CVD). The CVD system consisted of a furnace rolling around 48-inch long and 6-inch diameter quartz process tube and cooling fan, which allowed reducing the synthesis time by at least 2 h for each run/experiment. The furnace could be pre-heated up to the process temperature then moved towards the process zone. This would reduce the temperature ramp-up by 1 h. After CNT deposition, the furnace, which maintains the process temperature, was moved back to the outlet zone, and the cooling fan was used to drop the substrate temperature as fast as possible. This configuration allowed the process zone to cool down to room temperature within 10 min and saved about 1~2 h of time compared to conventional methods.

The substrates were set in a tubular CVD reactor (EasyTubeTM Nanofurnace system) for growth of vertically aligned Carbon Nanotubes. The CVD reactor was heated up to 700 °C under the flow of argon which created an inert environment. After reaching 700 °C, 200 sccm of hydrogen was introduced for 10 min in order to bring the catalyst back to its reduced state which is the active state. Then CVD was carried out by introducing the following gas mixtures into the reactor: ethylene (acetylene), hydrogen, and argon through a water bubbler (water vapor) with argon as a carrier for deposition. After that, the reactor was purged with argon during the final cooling step. The optimized synthesis parameters were:

Gases and flow rates: H_2_ 300 sccm; Ar + H_2_O 380 sccm; C_2_H_2_ 85 sccm; Ar 3500 sccm.

Temperature: 780 °C.

## 3. Results & Discussion

### 3.1. Fabrication of Patterned Catalyst System

**Figure 2:**
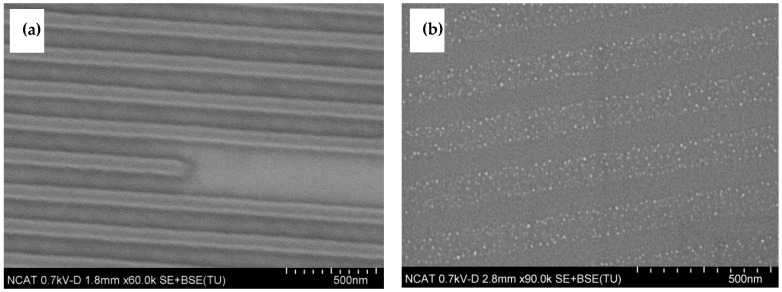
SEM images of imprinted substrate (**a**) and (**b**) catalyst pattern (Process A).

Figure 2 shows an example of a “negative” pattern transfer wherein the catalyst pattern was transferred with high precision. The sample was prepared with 100 nm grating stamp imprinted on PMMA resist on Si/SiO_2_ substrate and plasma-etched for 5 min at 100 W and 100 mTorr. Further, 10 nm alumina and 2 nm Fe were deposited using magnetron sputtering; and the lift-off was conducted in an ammonia-hydrogen peroxide solution and rinsed with acetone. In Process A show below, the alumina and Fe catalyst are deposited after NIL patterning which can save valuable catalyst materials and thereby prove more economical.

**Figure 3:**
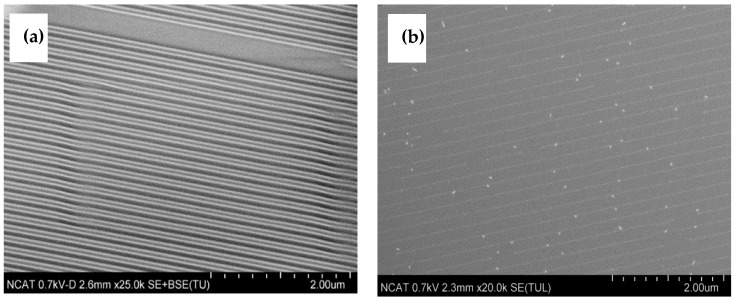
Imprinted substrate (**a**) and (**b**) catalyst pattern (Process B).

The example of a “positive” pattern of catalyst on a substrate is presented in Figure 3. It can be clearly seen that the catalyst lines have a thinner width than the original pattern; the width varies from 50 nm to 75 nm, as the period of pattern was 200 nm. The REI etching results in undercuts thereby, resulting in narrower catalyst patterning in Process B. The sample was prepared with 100 nm lines stamp imprinted on PMMA resist on the substrate with the 10 nm alumina and 2 nm Fe; plasma etched for 2 min at 100 W and 100 mTorr, then chemically etched with ceramic etchant (Sigma Aldrich) for 45 s and residue of PMMA was lifted-off with acetone.

### 3.2. NIL Process Parameters and Optimization

**Figure 4:**
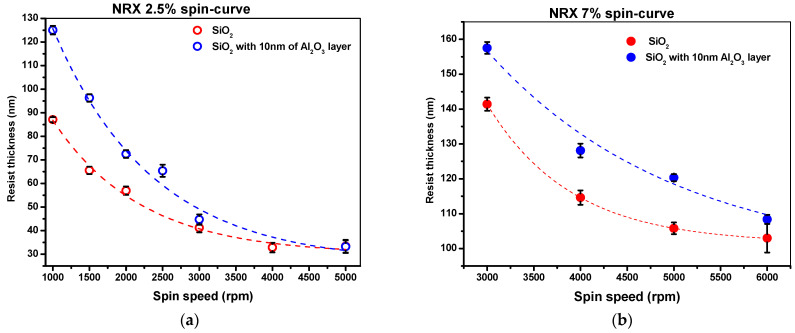
Spin-curve for NRX1025 (**a**) 2.5% and (**b**) 7% solution.

**Figure 5:**
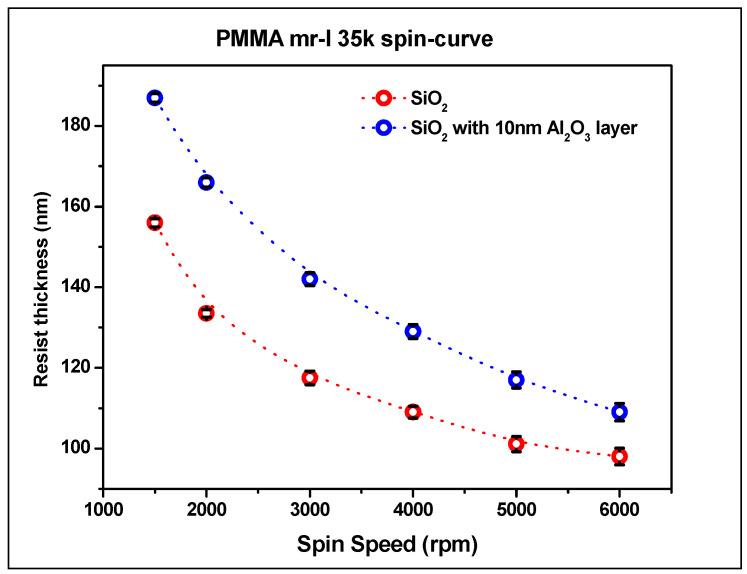
Spin-curve for PMMA mr-I 35k thermal resist.

The thermal mode of Nanonex 2000 nanoimprint lithographer was used to create a pattern on the substrate. The following thermal resist solutions were used: two solutions supplied by Nanonex—NRX1025 2.5% and 7%, and PMMA mr-35k from Sigma Aldrich. In order to determine the influence of the alumina layer on resist thickness, the resists NRX1025 2.5% and 7% and PMMA mr-I 35k were spin-coated on Si substrate and on 10 nm alumina layer deposited by RF sputtering with a roughness of Ra = 0.2 nm. The resists were spin-coated at various speeds from 2000 rpm to 6000 rpm, and the substrates were baked on a hot plate at 150 °C for 5 min to remove the residue of solvent. The thickness of the resist was measured by the XRD technique and profilometer. The spin-curves of NRX1025 2.5% and 7% solutions are presented in Figure 4 and Figure 5, respectively. Figure 4 presents the spin-curve for PMMA mr-35k thermal resist on pure Si substrate and on substrate with 10 nm of alumina layer.

As can be seen in Figure 4 and Figure 5, the deposition of alumina layer results in relatively higher resist thickness due to higher adhesion as compared to silicon substrate. The presence of an alumina layer increases the polymer thickness by 10–20%; however, with a higher spin speed, a thinner layer of thermal resist can be achieved. However, the uniformity of coating is better in the range of 2000–4000 rpm. The 2.5% NRX1025 resist can be spun down to a thickness of 40 nm, which is very important for imprinting with no residual layer.

### 3.3. Catalyst Deposition

A magnetron sputtering system was used for the deposition of an alumina support layer and an iron catalyst on Si/SiO_2_ substrates (Silicon Quest). Alumina was deposited as the first material, followed by Fe catalyst. Films were deposited from pure Al_2_O_3_ and Fe targets (purity 99.99%, Sputtering Ltd., Owatonna, MN, USA) using RF and DC power supply (100 W), respectively. The deposition was conducted at room temperature and at a working pressure of 1 mTorr. Pure argon was used as a carrier gas with a flow rate of 30 sccm. The flow rate of Ar gas was controlled using programmable gas flow controllers. The films’ thickness was controlled by deposition time. The deposition rates are presented in Table 1.

**Figure 6:**
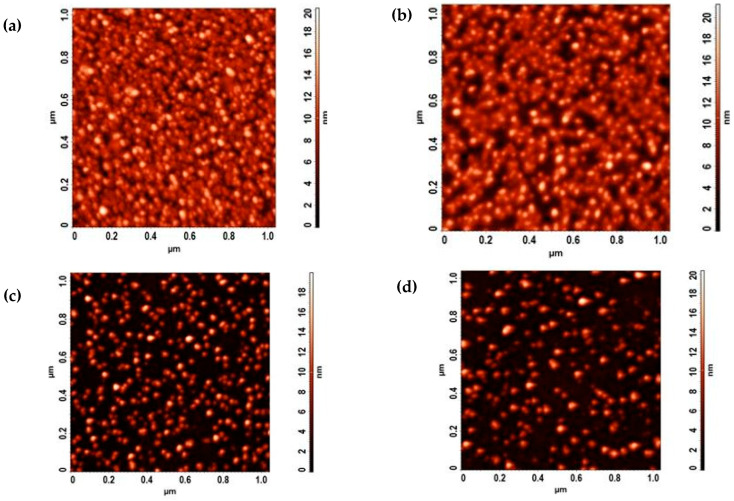
AFM study of catalyst deposition. DC sputtering at working pressures: (**a**) 1 mTorr, (**b**) 2 mTorr, (**c**) 4 mTorr, and (**d**) 6 mTorr, respectively.

The catalyst properties play a significant role in CVD synthesis of CNT. It is believed that catalyst size defines the diameter of CNT, and particle distribution is responsible for the density of the CNTs array [81,82]. In order to determine the effect of deposition pressure and substrate biasing on catalyst properties, an AFM study was conducted on Fe-Co catalyst deposited by DC sputtering on 10 nm thick alumina films. Figure 6 presents the AFM scans of catalyst samples made with the super-sharp NSG01_DLC tip. Particle analysis was conducted using NT-MDT Nova software (Version 1.0). The results show that lower deposition pressure resulted in closely seeded particles with a range of 6–12 nm.

### 3.4. Reactive Plasma Etching

**Figure 7:**
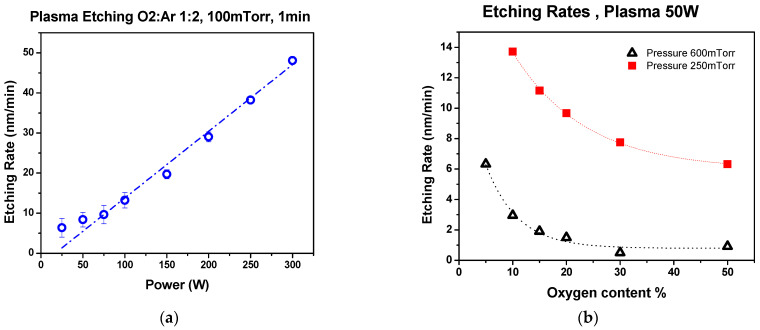
Plasma etching rates for NRX1025: (**a**) effect of plasma power, (**b**) effect of oxygen content.

**Figure 8:**
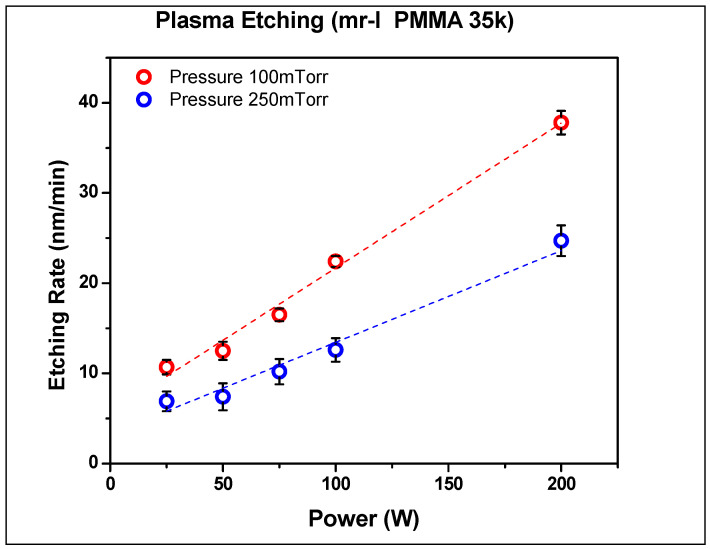
Plasma etching rates for PMMA.

The etching rates for thermal resist were determined by measuring the thickness of spin-coated films using XRD methods on the imprinted samples using AFM. The effects of pressure, applied power, and oxygen content in the reactive gas mixture were evaluated. As seen in Figure 7 and Figure 8, the etching rate of thermal resist is directly proportional to the applied power and inversely proportional to pressure. Increasing the oxygen content resulted in lower etching rates, as was observed by Hartney, Greene [83]. The PMMA resist has a lower etching rate than the NRX1025. These etching rates have an impact on the amount of resist being deposited during the spin coating stage for either negative or positive resist material types. Negative photoresists harden after exposure to UV light whereas positive photoresists soften after exposure to UV light [84]. Negative photoresists provide good adhesion to the substrate at a lower cost with shorter processing times. However, positive photoresists have higher resolution and thermal stability. In this research, we utilized thermal NIL in which both PMMA and NRX1025 resists hardened after crosslinking due to elevated temperatures.

**Figure 9:**
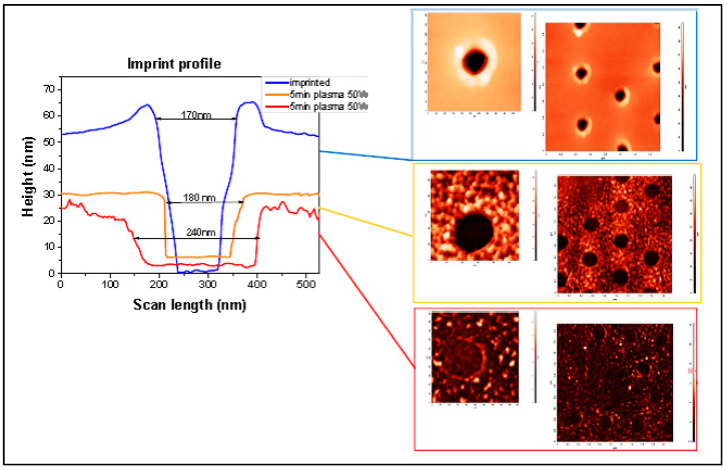
AFM study of plasma etching effect on thermal resist.

The AFM scans of imprinted and etched samples reveal the additional effect of the plasma etching process on the resist. As seen in Figure 9, the resist undergoes extra curing under plasma, wherein the polymer chain undergoes cross-linkage and becomes clearly visible. The over-curing and cross-linkage of resist were found undesirable for the lift-off process of further sample processing.

**Figure 10:**
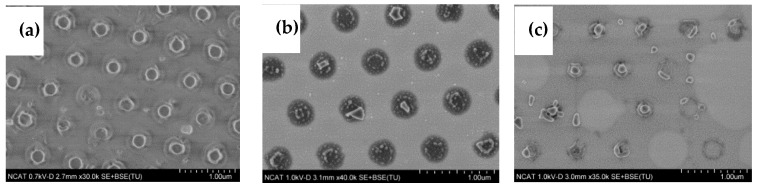
SEM study of plasma etching effect: (**a**) imprinted; (**b**) etched for 5 min; (**c**) etched for 8 min.

The AFM and SEM analyses of plasma-etched samples confirm that the process is unidirectional, and in order to maintain the desired planar size of the pattern, it should be minimized. The other undesirable effect of the plasma etching process was revealed by observing large areas of processed samples in the spots where the charge accumulated, and local charge breakthrough occurred (Figure 10c). To avoid the negative effects of the plasma process, the etching should be conducted at the lowest possible pressure and power for a short duration of time. Thus, near-zero residual layer imprint processes are highly desired.

### 3.5. CNT Patterns Grown by CVD

**Figure 11:**
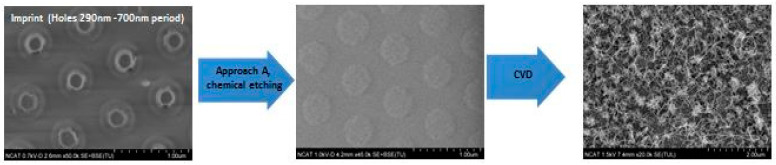
SEM images of short CNTs arrays fabricated by first approach (Process A) with the catalyst patterned by stamp with holes of 290 nm diameter.

**Figure 12:**
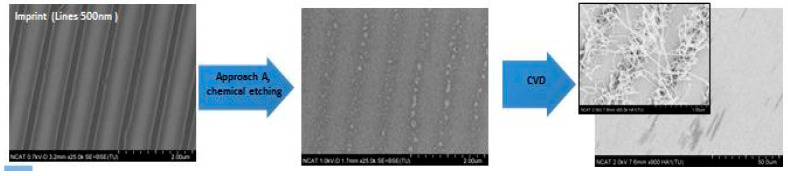
SEM images of short CNTs arrays fabricated by first approach (Process A) with the catalyst patterned by stamp with lines of 500 nm width.

**Figure 13:**
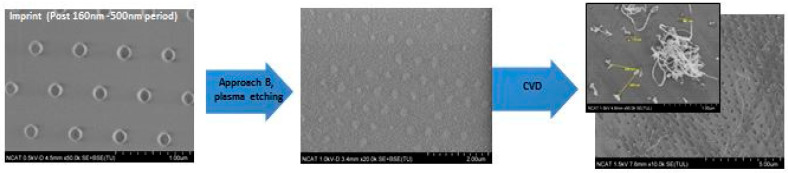
SEM images of short CNTs arrays fabricated by a second approach (Process B) with the catalyst patterned by stamp with dots of 160 nm diameter.

**Figure 14:**
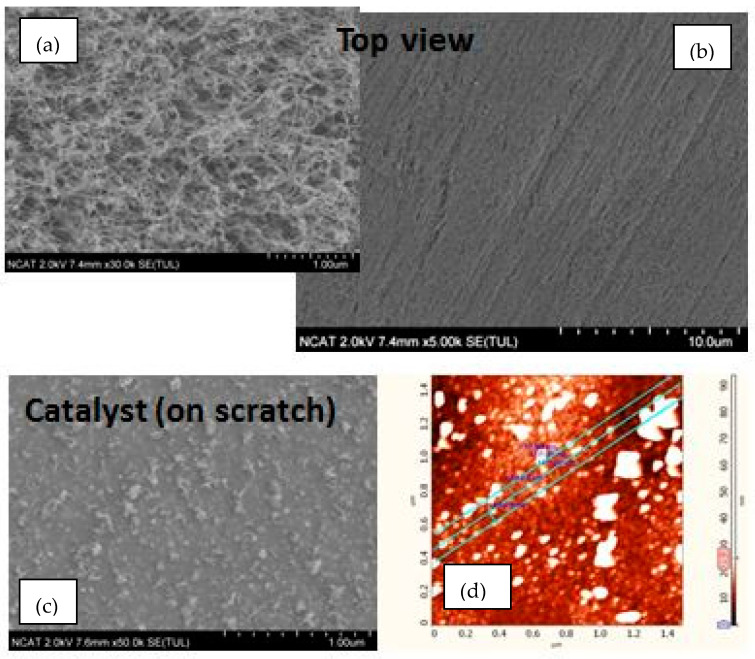
SEM images of long CNTs arrays fabricated by the first approach (Process A) with the catalyst patterned by stamp with lines of 70 nm width, 140 nm pattern period. Top view of a CNT arrays at high (**a**) and low (**b**) magnifications. Catalyst patterns under CNT arrays (**c**) SEM image and (**d**) AFM topography map.

The examples of CNT arrays grown for a short synthesis time (5 min) are presented in Figure 11, Figure 12 and Figure 13. Large areas of uniformly patterned CNT arrays were observed, which shows evidence that high-quality catalyst patterns were produced in a size range below 1 micron. The high-resolution images indicate that individual CNT pattern areas have grown at different rates: some are short and others are 10 times longer and curved without support from neighbors (inserted images on Figure 12 and Figure 13). This can explain why the wavy CNTs arrays were observed for long-term CVD synthesis and the difficulties in detecting the pattern of long CNT arrays with the small (below 100 nm) spacing of the pattern (Figure 14). The long-term CVD synthesis is related to longer chamber synthesis times of 20 min and longer, resulting in taller CNT growth.

**Figure 15:**
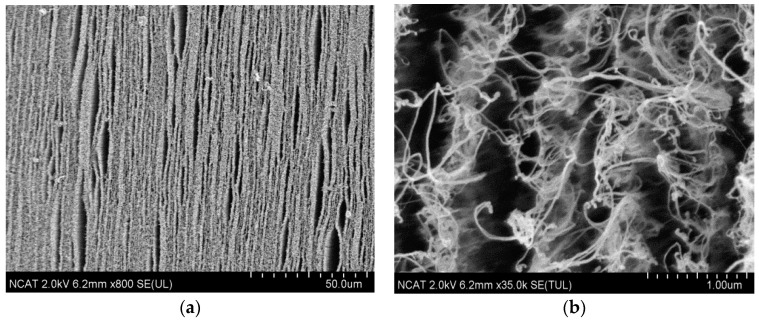
SEM images of long CNTs arrays fabricated by the second approach (Process B) with the catalyst patterned by stamp with lines of 455 nm width, 843 nm pattern period. Top view of a CNT arrays at (**a**) low and **(b**) high magnifications.

Figure 14 and Figure 15 show the linear CNT patterns grown by the two processes. CNTs were grown more than 100 microns in length in both cases. The narrower patterning in Process A is barely detectable (Figure 14b) due to smaller spacing between catalyst arrays, and “mushroom top” effect--the bending and entangling of the CNTs when the length/ diameter ratio became too high. This is also observed in Figure 15b which shows tangled CNTs. In order to confirm the presence of the catalyst pattern on the sample produced by Process A, the CNTs were removed, and the underlying surface was analyzed with the SEM (Figure 14c) and AFM (Figure 14d) to confirm the presence of a linear pattern with 70 nm spacing. The CNTs generated by Process B show a linearly arranged pattern of CNTs, as shown in Figure 15a.

### 3.6. CNT Characterization

**Figure 16:**
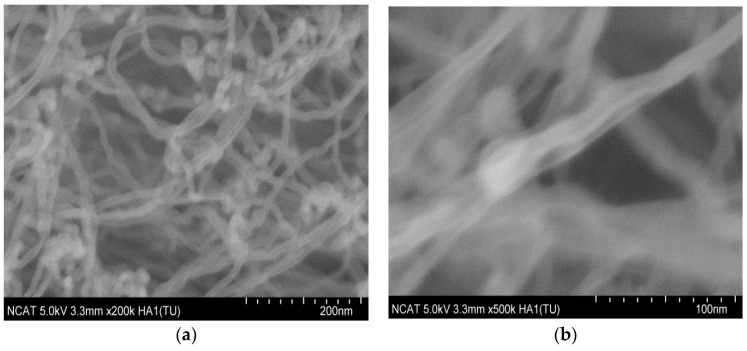
HR-SEM images of individual CNTs in array at (**a**) 200k and (**b**) 500k magnifications.

The SEM imaging and Raman analyses were used to characterize the patterned CNT arrays. The SEM images show the CNT distribution and quality of a pattern. It was also used to estimate the diameter of CNT and the straightness of arrays. Figure 16 presents the HR-SEM images of individual CNTs. It was observed that the individual CNTs have a diameter around 20 nm. The quality of CNTs depends on CVD synthesis parameters such as: gas compositions, flow rates, temperature, and catalyst preprocessing regime.

**Figure 17:**
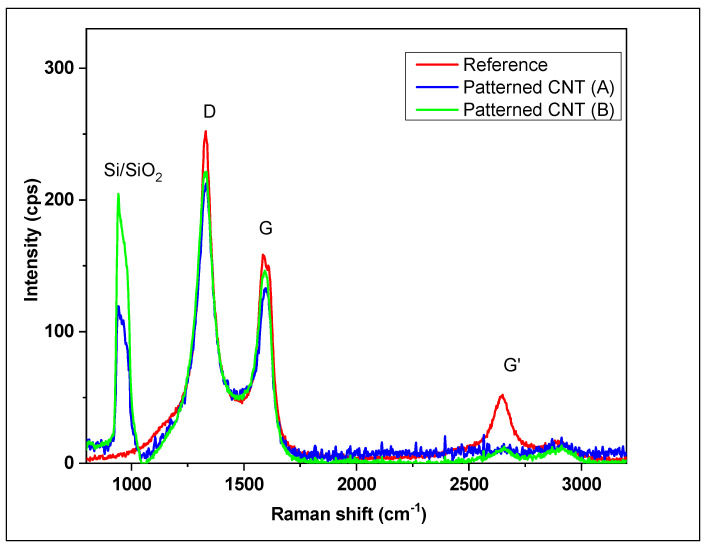
Raman spectra of reference and patterned CNT arrays.

The Raman spectra confirmed the results obtained by SEM analysis. As shown in Figure 17, the appearance of D and G bands are similar for both patterned and referenced samples. The ID/IG ratio varies from 1.3–1.5 which corresponds to the multiwall CNTs. There was no significant difference between the quality of patterned and reference samples. Table 2 shows a comparative analysis of D, G and G′ bands of the CNTs. The typical ID/IG ratio for MWCNT varies in a range 0.6–1.7, and lower ID/IG ratios reportedly indicates lower defects and higher quality MWCNTs [85]. The shift in a position of G′ band can be attributed to the presence of doping effects and/or strains induced during the fabrication process, and variations in the G′ mode position can provide information about the stacking arrangement and layer number of the graphene sheets in the nanotube structure [86]. In our case, we believe that G′ band shift indicates the strain results from mechanical stresses due to high tube length and lack of neighbors support on “empty” spaces of the pattern.

Pre-synthesized CNTs have been patterned by several methods including inkjet printing [87,88,89], screen printing [90,91,92], gravure printing [93], and microcontact printing [94,95,96]. However, these abovementioned approaches focus on alignment of CNTs for different applications, whereas our research focuses on patterning the catalyst to understand and control the growth mechanism of CNTs.

Casimirius et al. [97] provide a microcontact printing process for patterned growth of individual CNTs using a sol-gel precursor. A PDMS stamp was used for transfer of catalyst onto the silicon substrate creating features ranging from 10 to 100 µm. However, the authors noted that the softness of the stamp limited the fidelity of the printed features. Chatzikomis et al. [98] implemented inkjet printing to deposit iron salt as catalyst for CNT growth using CVD. They studied the effect of iron salt concentration on the rheology of ink and pattern quality. However, the inkjet-printed catalyst droplets formed a coffee-stain effect resulting in non-uniform CNT growth. Moreover, the feature resolution of patterning in inkjet printing ranges from 30 to 100 µm based on minimal nozzle sizes. Carpena-Núñez et al. have used electron beam lithography (EBL) to modify and pattern sapphire wafers down to the nanoscale [99]. They converted a ferrocene vapor precursor into Fe catalyst for patterned CNT growth. Similarly, Chen et al. focused ion beam (FIB) machining to etch silicon wafer with trenches. Further, they used vaporized iron phthalocyanine catalyst in the CVD process to grow CNTs from the patterned trenches [100]. Though the latter two methods were able to achieve nanoscale resolution, both EBL and FIB are extremely laborious and slow processes to create nanoscale patterns and limited to lab-based proof-of-concept solutions.

Our research, in contrast, focuses on the nanoscale patterning of catalyst for CNT growth based on two different process routes (Process A and B) using nanoimprint lithography. Nanoscale patterning reduces the catalyst deposition footprint to a few hundred nanometers. The reduced catalyst footprint aids in limiting the number of CNTs growing, resulting in less entanglement of the individual CNTs, permitting taller CNTs. Nanoimprint lithography is a highly scalable process for production of large-scale patterns. The NIL stamps can be reused hundreds of times to produce high-fidelity nanoscale patterns [101]. Thus, the methods implemented in this research enable scalable nanoscale patterning of CNT catalyst as a viable method for several industrial applications.

## 4. Conclusions

This research utilizes a combinatorial fabrication approach with nanoimprint lithography, magnetron sputtering and chemical vapor deposition techniques to fabricate CNTs. Process parameters were defined and optimized for each technology used in sample manufacturing. The deposition rates of alumina and catalyst metals were determined. The optimum working pressure was determined to produce smooth alumina. The effect of deposition pressure and substrate biasing on the distribution of catalyst particles was studied. The spin-curves and imprint temperature and time were evaluated for two thermal resist materials. The reactive plasma etching rates of thermal resists were determined. Catalyst patterns of lines, dots, and holes ranging from 70 nm to 500 nm were produced and characterized using AFM and SEM. Vertically aligned CNTs were successfully grown on a patterned catalyst using chemical vapor deposition. The produced CNTs arrays repeat the pattern of the catalyst and maintain the same quality as those synthesized on a regular catalyst substrate. This research shows that selecting the appropriate combination of technologies and maintaining the process controls at each step gives a possible solution to manipulating the shape of CNTs arrays, sizing them to the required dimensions, and placing them in a specified location. Our results will play a significant role in the fabrication of novel CNT-based devices for biosensors in the near future. Process A, which is the deposition of catalyst on pre-patterned substrate using NIL, is the recommended CNT growth process for biosensor application. Process A results in minimal alumina and FE catalyst deposition on the patterned substrate, thereby saving costs for expensive catalyst materials. The ultimate goal of further research will be to control individual CNT growth.

## Figures and Tables

**Table 1:** Deposition rates of catalyst materials.

Target	Power Source	Power(W)	Working Pressure (mTorr)	Deposition Rate (nm/min)
Al_2_O_3_	RF	100	1	2.0
Al_2_O_3_	RF	100	4	1.8
Al_2_O_3_	RF	250	1	6.1
Al_2_O_3_	RF	250	2	5.5
Al_2_O_3_	RF	250	4	5.0
Al_2_O_3_	RF	250	6	4.6
Fe	DC	100	1	4.8
Fe	DC	100	4	4.5
Co	DC	100	1	5.2
Co	DC	100	4	4.9

**Table 2:** Comparative analysis of bands in patterned CNT arrays.

CNT Type	D (cm^−1^)	G (cm^−1^)	G′ (cm^−1^)	ID/IG Ratio
Reference MWCNT	1329	1594	2643	1.36
Patterned CNT (A)	1328	1594	2656	1.42
Patterned CNT (B)	1327	1592	2648	1.43

## Data Availability

The data presented in this study are available on request from the corresponding author.
